# Biomechanical Assessment of the Validity of Sheep as a Preclinical Model for Testing Mandibular Fracture Fixation Devices

**DOI:** 10.3389/fbioe.2021.672176

**Published:** 2021-05-06

**Authors:** Vincenzo Orassi, Georg N. Duda, Max Heiland, Heilwig Fischer, Carsten Rendenbach, Sara Checa

**Affiliations:** ^1^Julius Wolff Institute, Charité – Universitätsmedizin, Corporate Member of Freie Universität Berlin, Humboldt-Universität zu Berlin and Berlin Institute of Health, Berlin, Germany; ^2^Department of Oral and Maxillofacial Surgery, Charité – Universitätsmedizin, Corporate Member of Freie Universität Berlin, Humboldt-Universität zu Berlin and Berlin Institute of Health, Berlin, Germany; ^3^Berlin-Brandenburg School for Regenerative Therapies, Berlin, Germany

**Keywords:** mandible fracture, fracture fixation, finite element, sheep mandible, mechanobiology, biomechanics

## Abstract

Mandibular fracture fixation and reconstruction are usually performed using titanium plates and screws, however, there is a need to improve current fixation techniques. Animal models represent an important step for the testing of new designs and materials. However, the validity of those preclinical models in terms of implant biomechanics remains largely unknown. In this study, we investigate the biomechanics of the sheep mandible as a preclinical model for testing the mechanical strength of fixation devices and the biomechanical environment induced on mandibular fractures. We aimed to assess the comparability of the biomechanical conditions in the sheep mandible as a preclinical model for human applications of fracture fixation devices and empower analyses of the effect of such defined mechanical conditions on bone healing outcome. We developed 3D finite element models of the human and sheep mandibles simulating physiological muscular loads and three different clenching tasks (intercuspal, incisal, and unilateral). Furthermore, we simulated fractures in the human mandibular body, sheep mandibular body, and sheep mandibular diastema fixated with clinically used titanium miniplates and screws. We compared, at the power stroke of mastication, the biomechanical environment (1) in the healthy mandibular body and (2) at the fracture sites, and (3) the mechanical solicitation of the implants as well as the mechanical conditions for bone healing in such cases. In the healthy mandibles, the sheep mandibular body showed lower mechanical strains compared to the human mandibular body. In the fractured mandibles, strains within a fracture gap in sheep were generally not comparable to humans, while similar or lower mechanical solicitation of the fixation devices was found between the human mandibular body fracture and the sheep mandibular diastema fracture scenarios. We, therefore, conclude that the mechanical environments of mandibular fractures in humans and sheep differ and our analyses suggest that the sheep mandibular bone should be carefully re-considered as a model system to study the effect of fixation devices on the healing outcome. In our analyses, the sheep mandibular diastema showed similar mechanical conditions for fracture fixation devices to those in humans.

## Introduction

Fixation with plates and screws at the mandible is used as gold-standard in patients with fractures (Champy et al., 1978; Sauerbier et al., 2008; Munante-Cardenas et al., 2015) and segmental resections due to tumors or osteonecrosis (Evans et al., 1995; Shaw et al., 2004; Isler et al., 2018; Rendenbach et al., 2018). Mandible fractures account for around 50% of all maxillofacial injuries (Odom and Snyder-Warwick, 2016) and may compromise mandibular mechanics, function, and facial esthetics. Early mobilization is needed and requires adequate stability at the fracture site. Further, these conditions may be optimized to promote healing (Claes et al., 1997; Augat et al., 2003). Thus, knowledge of the biomechanical environment in the mandible as a result of the activity of the masticatory muscles is essential to guarantee an optimal fixation and to maintain the reduction. In clinical practice, standard fixation of simple fractures is performed through load-sharing devices, which is usually achieved with titanium miniplates and monocortical screws along Champy’s ideal lines of osteosynthesis (Champy et al., 1978). The latter helps to reduce the empiricism behind the choice of plate positioning, neutralizing the shear strains exerted at the fracture site, and restoring the physiological strain patterns in the bone tissue. Besides fracture management, load-sharing osteosynthesis is also used for the fixation of free flaps at the mandible following continuity resection due to benign or malign tumors, osteoradionecrosis, and medication-related osteonecrosis of the jaws (Shaw et al., 2004; Robey et al., 2008; Liu et al., 2016). Despite high success rates in both maxillofacial trauma and reconstructive surgery, fixation-related complications remain. These include material failure, non-union, and plate-related infections (Shaw et al., 2004; Robey et al., 2008; Seemann et al., 2010; van den Bergh et al., 2012; Gutta et al., 2014; Liu et al., 2016; Rendenbach et al., 2019). To improve current treatment options and to develop and test new approaches, including innovative implant materials, e.g., magnesium alloys (Byun et al., 2020), profound basic research is necessary.

For this reason, animal models are often used to test novel treatment strategies. In the maxillofacial field, non-human primates are considered the most appropriate model for human bone (Hylander, 1979; El Deeb et al., 1985; Bolly et al., 2019), however, the ethical implications and handling difficulties make them a non-viable solution. Similar limitations can be found with companion animals, like cats and dogs, while minipigs, which are currently one of the most used animal models for craniomaxillofacial studies, despite similarities to humans in the biomechanics of mastication (Vapniarsky et al., 2017, 2018), present high stiffness of both mandibular bone and soft tissues. In the last decades, sheep have been used to test fixation devices for fractures (Tepic et al., 1997; Hente et al., 1999; Krischak et al., 2002; Schell et al., 2005; Claes et al., 2008) and large bone defects (den Boer et al., 1999; Egermann et al., 2008; Christou et al., 2014; Pobloth et al., 2018) in long bones. In the mandible, from the surgical point of view, sheep bring important advantages in terms of soft tissue management and body weight comparable to humans. However, differences between human and sheep mandible must be taken into consideration. Major anatomical dissimilarities include the absence of the upper incisor teeth, the mandibular body length, and the presence of a toothless region (diastema). Moreover, compared to humans, the presence of flat condylar surfaces and the different radius of the curve of Spee alter the biomechanics of mastication, inducing masticatory movements that result in predominant compressive and translational biting, in contrast to the rotational movements typical of carnivores, and consistent with a feeding strategy that requires shear forces at the occlusion to break the stiff grass fibers (Popowics and Herring, 2006; Watson et al., 2018). Although muscles involved in mastication are similar in humans and sheep, they do present differences in muscular attachments and proportions (Barone, 1980). At the microscopic level, sheep present a mainly primary bone structure in contrast to the predominantly secondary one in humans (Pearce et al., 2007). Although sheep lack secondary osteons, they generally proved to be a good model mimicking human bone biology and healing in long bones (Chavassieux et al., 1991; den Boer et al., 1999; Pearce et al., 2007). However, potential differences in biomechanics and mechanobiology make it questionable if this also holds true for the mandible.

Several biomechanical studies used *in vivo* sheep models to evaluate fixation system stability and its influence on the healing outcome in the mandible. For example, Poon and Verco (2013) compared the results of locking and conventional miniplates on angled fracture healing, observing an improved outcome for the formers. Similarly, Gutwald et al. (2011) performed a step-like osteotomy in the sheep mandibular diastema treated with different fixation devices, reporting, after 8 weeks, an advanced ossification for locking miniplates. In the same region, Schouman et al. (2016) observed how bone ingrowth is enhanced by a low-stiffness titanium porous scaffold in a large mandibular defect. Rasse et al. (2007) tested new biodegradable plates to treat condylar fractures, obtaining promising outcomes. Moreover, sheep have been reconsidered as a good preclinical model for the temporomandibular joints (TMJs), thanks to morphological and biomechanical similarities to humans found in the TMJ discs (Ângelo et al., 2016; Almarza et al., 2018). Besides, mechanical tests on *ex vivo* sheep mandibles have been also performed to evaluate plate resistance to cycling loading (Pituru et al., 2016), the influence of the osteotomy angle on fixation stability (Pektas et al., 2012), and to verify the reliability of new fixation techniques (de Olivera et al., 2012).

Furthermore, in the last decades, computational modeling has been largely used for biomechanical analyses in the human mandible (Korioth et al., 1992; Korioth and Hannam, 1994; Vollmer et al., 2000; Lovald et al., 2006, 2010; Vajgel et al., 2013; Commisso et al., 2015; Huo et al., 2015; Gutwald et al., 2017; Liu et al., 2017; Luo et al., 2017). In particular, finite element (FE) analysis has been performed to calculate stress and strain values within the fixation systems and mandibular bone, otherwise hard to obtain with *in vivo* or *in vitro* experiments. However, to our knowledge, only one study developed a finite element model (FEM) of the whole sheep mandible, in two dimensions, correlating the stress distribution within the bone tissues to the mandibular morphology (De Jongh et al., 1989). Consequently, a 3D FE computer model of the whole sheep mandible in healthy and fractured conditions is still missing. To date, it remains unknown if the biomechanical boundary conditions of a fracture in a sheep mandible are generally comparable with those in humans.

This study aimed to investigate whether sheep can be considered a valid large animal model for preclinical testing of human fracture fixation devices in the mandible by comparing stresses within the implants and tissue straining of the healing zone in sheep and humans. Since the mechanical conditions within the healing region are known to influence the healing outcome (Claes et al., 1997), a comparison between the local biomechanical environment of the healing zone after fracturing in sheep and human mandibles would allow judging on the appropriateness of such comparisons.

## Materials and Methods

3D FE computer models of fully dentate human and sheep mandibles belonging, respectively, to a 60-year-old woman and an 18-month-old female sheep of the species *Ovis aries*, both in healthy conditions, were reconstructed from Computed Tomography (CT) scans. The CT scans were performed in axial mode, with a slide thickness of 0.4 mm (ProMax, Planmeca, Finland) in humans, and in helical mode, with a slide thickness of 0.6 mm (LightSpeed VCT, General Electric, United States) in sheep. DICOM images resulting from the scans were imported into the commercial software Amira 6.0.1 (Zuse Institute Berlin, Germany), where cortical and trabecular bone tissues were labeled, through automatic and manual segmentation tools based on the gray-scale values, and meshed, using linear tetrahedral elements (element type C3D4). The models were then imported into the commercial finite element software Abaqus/CAE v.6.18 (Dassault Systèmes Simulia Corp., United States), where the linear mesh was converted into a quadratic mesh (element type C3D10) and the model parameters were defined.

### Loading and Boundary Conditions

**FIGURE 1:**
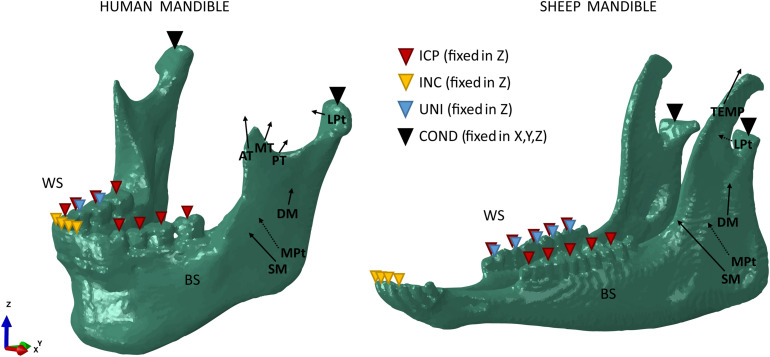
Loading and boundary conditions for the human (left) and sheep (right) mandibles during intercuspal (ICP), incisal (INC), and unilateral (UNI) clenching. Bite force was simulated restraining the vertical displacement at the occlusion and both the human condyles and the sheep condylar processes (COND) in the 6 degrees of freedom. During UNI clenching, the right mandibular body is considered as the working side (WS) and the left mandibular body as the balancing side (BS).

For both the human and sheep models, three different clenching tasks were simulated in intercuspal (ICP), incisal (INC), and right unilateral (UNI) biting (Figure 1). To simulate occlusion, vertical displacement was not allowed for different teeth groups: all molars and premolars teeth (ICP), all incisors teeth (INC), and first molar/second premolar teeth (UNI). The human condyles and the sheep condylar processes (COND) were assumed locked in the glenoid fossa and in the mandibular fossa, respectively, and thus they were restrained from movement in all six degrees of freedom.

The main closing muscle groups were simulated to reproduce a maximum bite force condition at the occlusal plane. The muscles include superficial masseter (SM), deep masseter (DM), anterior temporalis (AT), medial temporalis (MT), posterior temporalis (PT), medial pterygoid (MPt), and inferior lateral pterygoid (LPt).

In the human mandible, previous studies’ outcomes (Korioth et al., 1992; Korioth and Hannam, 1994) combined with anatomical observation allowed to define attachment area, orientation, and force magnitude of each muscle group (Figure 1). In the sheep mandible, mandibular muscle forces have not been completely reported, therefore the same approach used by Nelson (1986) was chosen to estimate the force magnitude of each muscle group. Physiological cross-sectional areas (PCSAs) of the muscles were estimated from a Magnetic Resonance Imaging (MRI) scan (3T Skyra, Siemens, Germany) of the sheep head, as the ratio between muscle volume and muscular fibers length (Eq. 1) (Weijs and Hillen, 1984), both calculated in Amira. PCSAs were then used to calculate the magnitude of muscular forces (F) based on Eq. 2 (Nelson, 1986).

(1) $$\mathrm{PCSA}=\frac{\mathrm{Muscle}\,\mathrm{volume}}{\mathrm{Fiber}\,\mathrm{length}}$$

(2) $$F=A^{*}\,K^{*}\,\mathrm{PCSA}$$

with K (N/cm^2^) as a musculoskeletal constant and A (−) as the fiber activation ratio (Table 1). When A = 1.0, all the fibers are activated and the muscle force is equivalent to the maximum force that can be exerted by the specific muscle group.

**TABLE 1:** Sheep maximum muscle forces were obtained from the PCSAs, assuming the musculoskeletal constant K = 40 (N/cm^2^) and the fiber activation A = 1.0 (Eq. 2) (Nelson, 1986).

**Sheep mandible muscle**	**Muscle volume (cm^3^)**	**Fiber length (cm)**	**PCSA (cm^2^)**	**Musculoskeletal constant K (N/cm^2^)**	**Maximum muscle force (N)**
Superficial masseter	87.1	11.911	7.313	40	292.5
Deep masseter	12.4	3.636	3.410	40	136.4
Temporalis	40.8	9.837	4.148	40	165.9
Medial pterygoid	42.8	10.547	4.068	40	162.7
Lateral pterygoid	4.7	2.993	1.570	40	62.8

*PCSA, physiological cross-sectional area.*

The muscle activation patterns for a specific clenching task have been described by Korioth et al. (1992) and were used in this study, in both the human (Table 2) and sheep (Table 3) models. In sheep, the temporalis muscle (TEMP) was modeled as a whole, due to the uncertainty in distinguishing the three groups. The activation constant for the temporalis muscle was obtained as the mean value of the human anterior, medial, and posterior temporalis constants. The XY was defined as the transverse plane, XZ as the coronal plane, and YZ as the sagittal plane.

**TABLE 2:** Muscle groups’ maximum force, direction cosines, and activation patterns, based on the specific clenching tasks in the human mandible.

**Human mandible muscle**	**Maximum muscle force (N)**	**Direction cosine**				**Fiber activation A (−)**					
		**X**		**Y**	**Z**	**ICP**		**INC**		**UNI**	
		**Right**	**Left**			**Right**	**Left**	**Right**	**Left**	**Right**	**Left**
Superficial Masseter	190.4	−0.207	0.207	−0.419	0.884	1.0	1.0	0.40	0.40	0.72	0.60
Deep Masseter	81.6	−0.546	0.546	0.358	0.758	1.0	1.0	0.26	0.26	0.72	0.60
Anterior Temporalis	158.0	−0.149	0.149	−0.044	0.988	0.98	0.98	0.08	0.08	0.73	0.58
Medial Temporalis	95.6	−0.222	0.222	0.500	0.837	0.96	0.96	0.06	0.06	0.66	0.67
Posterior Temporalis	75.6	−0.208	0.208	0.855	0.474	0.94	0.94	0.04	0.04	0.59	0.39
Medial Pterygoid	174.8	0.486	−0.486	−0.373	0.791	0.76	0.76	0.78	0.78	0.84	0.60
Lateral Pterygoid	66.9	0.630	−0.630	−0.757	−0.174	0.27	0.27	0.71	0.71	0.30	0.65

*The muscular force components (Fx, Fy, and Fz) were obtained by multiplying the maximum force magnitude by the direction cosines, and the fiber activation value (Korioth et al., 1992). ICP, intercuspal; INC, incisal; UNI, unilateral.*

**TABLE 3:** Muscle groups’ maximum force, direction cosines, and activation patterns, based on the specific clenching tasks in the sheep mandible.

**Sheep mandible muscle**	**Maximum muscle force (N)**	**Direction cosine**				**Fiber activation A (−)**					
		**X**		**Y**	**Z**	**ICP**		**INC**		**UNI**	
		**Right**	**Left**			**Right**	**Left**	**Right**	**Left**	**Right**	**Left**
Superficial masseter	292.5	−0.054	0.054	−0.840	0.539	1.0	1.0	0.40	0.40	0.72	0.60
Deep masseter	136.4	−0.539	0.539	−0.248	0.805	1.0	1.0	0.26	0.26	0.72	0.60
Temporalis	165.9	0.036	−0.036	0.840	0.541	0.96	0.96	0.06	0.06	0.66	0.55
Medial pterygoid	162.7	0.186	−0.186	−0.083	0.979	0.76	0.76	0.78	0.78	0.84	0.60
Lateral pterygoid	62.8	0.400	−0.400	−0.716	0.574	0.27	0.27	0.71	0.71	0.30	0.65

*The muscular force components (Fx, Fy, and Fz) are obtained by multiplying the maximum force magnitude by the direction cosines, and the fiber activation value. ICP, intercuspal; INC, incisal; UNI, unilateral.*

### Fracture Fixation

**FIGURE 2:**
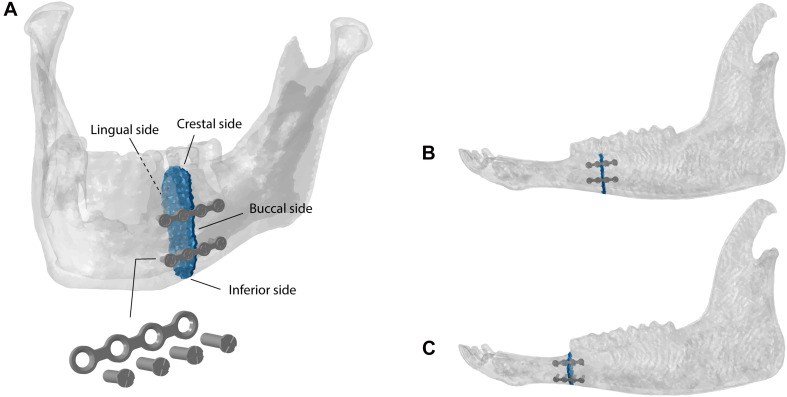
Fractures and fixation devices in the **(A)** human and **(B)** sheep mandibular bodies and in the **(C)** sheep mandibular diastema.

Simple fractures were simulated in the human (Figure 2A) and sheep (Figure 2B) left mandibular bodies and sheep left mandibular diastema (Figure 2C), defining a transverse element-set between the last premolar and the first molar teeth, for the former cases, and at the distal end of the mandibular diastema, for the latter case. The fracture gap was approximately 1.5 mm in width, in all models. According to AO’s guidelines (Ao Foundation, 2021) and Champy’s principles (Champy et al., 1978), the fractures were fixated with two parallel 4-hole 2.0 miniplates 1 mm thick and simplified (no thread) monocortical screws, 7 mm long (Figure 2A). The fixation devices were designed using the 3D-CAD software SolidWorks 2019 (Dassault Systèmes, France), based on commercially available devices. The miniplates were then positioned on the mandibles, through bending and torsional manipulations, to reach a good adaptation to the bone surface, following clinical advice. Afterward, miniplates and screws were imported into Abaqus, preserving the position, and then meshed with quadratic tetrahedral elements (C3D10). Tie constraints were defined between plates and screws, and between screws and underlying bone tissues.

### Material Properties

All materials were considered isotropic, homogeneous, and linear elastic. In the human model, Young’s moduli of cortical and trabecular bone were taken as 15,000 MPa and 300 MPa, comparable with the experimental values calculated, respectively, by Schwartz-Dabney and Dechow (2003) and Lakatos et al. (2014). In the sheep model, the material properties used in this study are based on the work of Li et al. (2013), with Young’s moduli of 15,750 MPa and 300 MPa for cortical and trabecular bone, respectively. For all bone tissues, a Poisson’s ratio of 0.30 was chosen.

In the fractured mandibles, granulation tissue properties were assigned to the elements within the fracture line, with Young’s modulus of 1 MPa and Poisson’s ratio of 0.30, to simulate the mechanical conditions at the initial phase of healing (Leong and Morgan, 2008). Titanium alloy Ti-6Al-4V material properties were assigned to the osteosynthesis devices, with Young’s modulus of 110,000 MPa and a Poisson’s ratio of 0.34 (MatWeb, 2021). The titanium yield strength was considered equal to 880 MPa (MatWeb, 2021) and was used as a threshold for the fixation device failure prediction.

### Mesh Convergence Study

A mesh convergence test was performed comparing four different mesh sizes. In the human mandible, the number of tetrahedrons varied from mesh (A) 1,099,247 elements (finest mesh), mesh (B) 645,150, mesh (C) 288,967, to mesh (D) 168,784 (coarsest mesh). In the sheep mandible, the mesh size varied from (A) 1,395,528 elements (finest mesh), (B) 825,803, (C) 488,786, and (D) 354,805 (coarsest mesh). The different models were then imported into ABAQUS, where the linear mesh was converted into a quadratic mesh (element type C3D10). Simplified loading conditions were applied, simulating loads with components (Fx = 0, Fy = −50 N, Fz = 50 N), distributed on a surface of a 10 mm radius at the masseter attachments, to remove any variability introduced by the muscle attachments. The mandibular bodies were chosen as regions of interest and averaged von Mises stresses and maximum and minimum principal strains, for each mesh size, were calculated and compared to the finest mesh outcomes. In both cases, mesh B was a good compromise between accuracy (relative error <5%) and computational costs, and was therefore chosen as the definitive mesh size.

## Results

### Mechanical Strains in the Healthy Mandibles

In the human mandible, maximum bite force values at the occlusal planes for the ICP, INC, and UNI clenching tasks were about 450 N, 180 N, and 500 N, respectively. In the sheep model, the maximum bite force resulted generally smaller than in humans. The ICP, INC, and UNI clenching tasks resulted, at the occlusion, in a maximum bite force of about 400 N, 80 N, and 400 N, respectively.

**FIGURE 3:**
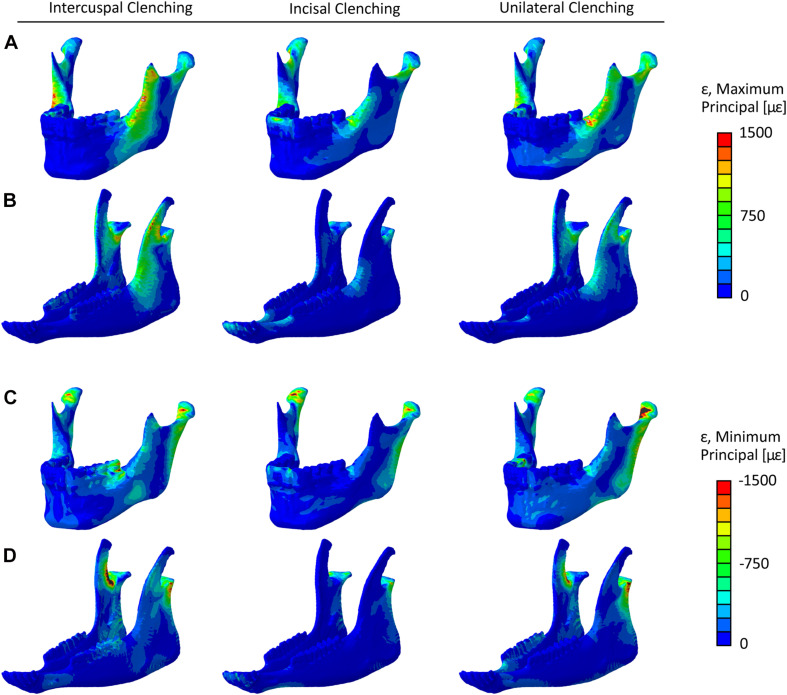
Maximum principal strain (ε) distribution in the healthy **(A)** human and **(B)** sheep mandibles, and minimum principal strain distribution in the healthy **(C)** human and **(D)** sheep mandibles, for the intercuspal (ICP), incisal (INC), and unilateral (UNI) clenching tasks.

Figure 3 shows each clenching task’s effect on the strain distribution and magnitude within the mandibular bone in the human and sheep healthy models. In both models, higher strains were predicted in the rami, coronoid processes, and condylar necks with tensile and compressive strains up to 1,200 με and −1,750 με, respectively. Compressive strains up to 1,500 με were also predicted during ICP and UNI tasks at the molar region.

**FIGURE 4:**
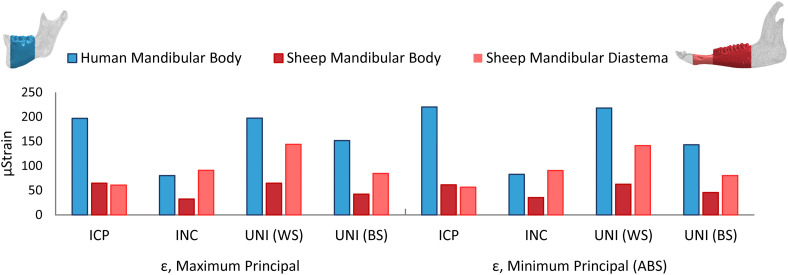
Average maximum and minimum principal strains (ε) in the healthy human and sheep mandibular body, and in the sheep mandibular diastema, for the intercuspal (ICP), incisal (INC), and unilateral (UNI) clenching tasks, the latter in both working (WS) and balancing sides (BS).

Since the mandibular body was chosen for the simulation of a simple fracture and the application of the fixation devices, the average maximum and minimum principal strain within the human and sheep mandibular body and the sheep mandibular diastema (Figure 4) were calculated. For almost all the clenching tasks, the mechanical strains were considerably lower in the sheep mandibular bone compared with the human (Figure 4). Interestingly, in the sheep mandibular diastema, the strains were higher than in the sheep mandibular body and closer to the strains found in the human mandibular body. Particularly, during the INC task, mechanical strains in the sheep mandibular diastema were the closest to those predicted in the human mandibular body.

### Mechanical Strains Within the Fracture Gap

**FIGURE 5:**
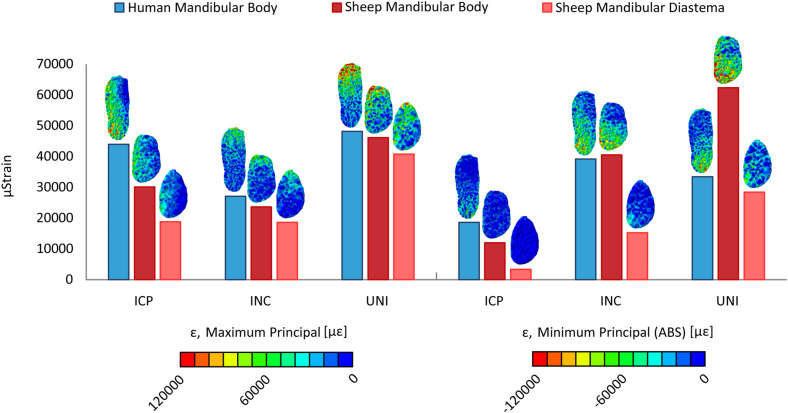
Distribution and average values of the maximum and minimum principal strains (ε) within the fracture gap, in the human and sheep models, for the intercuspal (ICP), incisal (INC), and unilateral (UNI) clenching tasks.

Figure 5 shows the averaged maximum and minimum principal strains within the fracture gap in the human and sheep models. Principal strain distributions show that, in both models, the crestal side is mostly under tension, while the inferior side is mainly under compression (Figure 5). In addition, the lingual side is more mechanically solicited than the buccal side, where the miniplates are fixated.

The strain values within the sheep and human mandibular body fractures are generally closer to each other, compared to the mandibular diastema fracture (Figure 5). Major differences are found during the UNI task, where the minimum principal strain within the sheep mandibular body fracture is much higher compared to the human mandibular body fracture, as well as during the ICP task, where the mechanical solicitation in the sheep fractures is remarkably lower than in humans. For all loading cases, the mechanical strains, both in tension and compression are considerably lower within the sheep mandibular diastema fracture compared with the human mandibular body fracture.

### Von Mises Stresses Within the Fixation Devices

**FIGURE 6:**
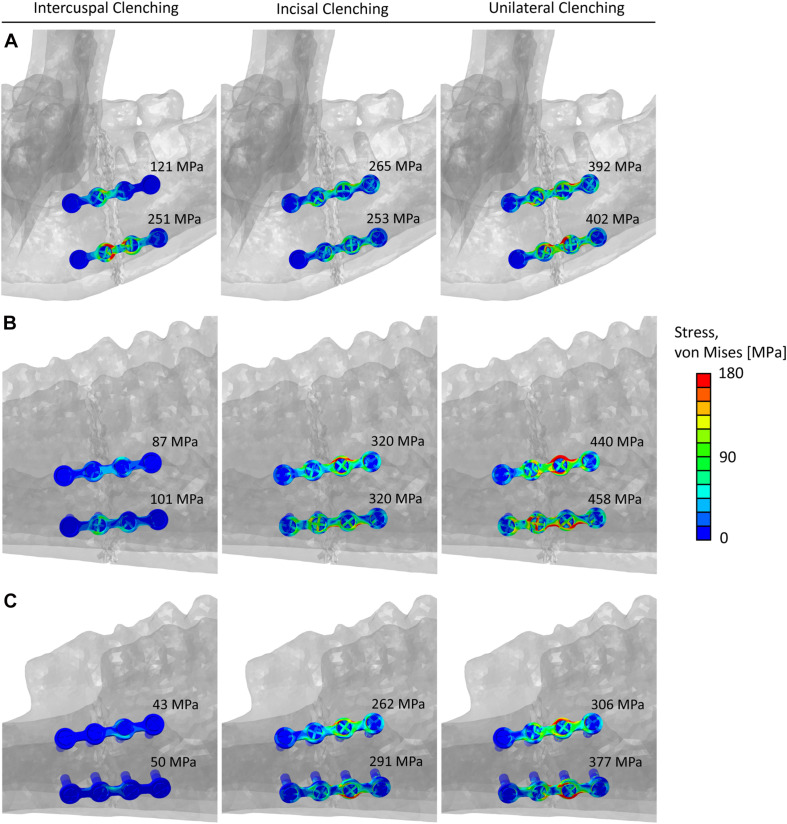
Von Mises stresses distribution and peak values within the miniplates and screws in the **(A)** human mandibular body fracture, **(B)** sheep mandibular body fracture, and **(C)** sheep mandibular diastema fracture, for the intercuspal (ICP), incisal (INC), and unilateral (UNI) clenching tasks.

Figure 6 shows the von Mises stress distribution in the fixation devices for the three analyzed fracture scenarios and the three clenching tasks. Generally, the miniplates were highly mechanically solicited in the proximity of the fracture line, and in none of the cases, von Mises stresses exceeded the titanium yield strength.

To perform a comparison of the von Mises stresses within the implants, first, the 0.1% of the upper higher stress values were excluded from the calculation to remove stress singularities due to the applied constraints between screws and miniplates. Subsequently, the peak von Mises Stress was calculated by averaging the ten highest stress values, in both the top and bottom implants (Figure 6).

## Discussion

Osteosynthesis for mandibular fractures is a highly investigated topic in maxillofacial research. The fixation methods must provide at the fracture site adequate stability, restoring the physiological biomechanical conditions to promote bone healing and thus provide rapid recovery of the patient. However, several complications remain and thus make necessary a constant improvement of the current fixation systems and the development of new treatment strategies. For example, the development of novel biodegradable fixation devices requires not only *in vitro* but also *in vivo* evaluation of the biomechanical and biological interactions with the living tissues. Preclinical testing is, therefore, essential to keep up with the continuous innovation in the field and the choice of an appropriate animal model is crucial to biomechanically evaluate the performance of the osteosynthesis devices and the different materials under analysis. In this study, we investigated sheep mandible as an animal model for testing fixation devices and their potential influence on the healing outcome. We built 3D finite element models of the human and sheep mandibles to biomechanically test clinically used fixation devices under different clenching tasks and different fracture scenarios. We compared the results in terms of stresses within the fixation devices and strains within the healthy mandibular bone tissues and at the fracture sites. The former provides evidence of possible hardware overloading and, eventually, failure, while the latter is known to guide the bone healing process (Claes et al., 1997). We found that the biomechanical environment at the fracture site presents notable differences between the human and sheep mandibles. We also found that the sheep mandibular diastema region seems to be a good candidate for testing fixation devices, while the sheep mandibular body region may present higher risks of hardware failure for specific biting tasks.

Several studies have developed finite element models of the healthy human mandible. The strain distribution and magnitudes predicted in this study are in agreement with those studies (Korioth et al., 1992; Baek et al., 2012). In particular, our model predicted average strains in the range between 75 με and 200 με in the mandibular body, which are similar to those predicted by Baek et al. (2012), during ICP clenching, and by Korioth et al. (1992), during the UNI clenching. Moreover, the maximum bite forces at the occlusion are consistent with previous studies (Korioth et al., 1992; Paphangkorakit and Osborn, 1997; Bakke, 2006; Varga et al., 2011).

To our knowledge, this is the first study to develop a 3D finite element model of the whole sheep mandible. De Jongh et al. (1989) previously developed a 2D sheep mandible finite element model, however, their predictions did not take into account the complex three-dimensional geometry of the sheep mandible. Schmitt et al. (2016) used a 2D computational model to describe the scaffold-guided bone healing process in the sheep mandibular diastema. While Freddo et al. (2014) developed a 3D finite element model of the sheep mandibular angle region during distraction osteogenesis, although with simplified loading and boundary conditions.

Finite element predictions in this study show that the healthy sheep mandible has a different biomechanical behavior compared to the human mandible, assuming the same clenching tasks. Strong vertical components of the muscle forces together with the condylar and occlusal reaction forces cause a sagittal bending of the mandibular body during ICP, INC, and UNI clenching, for the latter in the balancing side, and a combination of sagittal bending and torsion of the working side during UNI clenching (van Eijden, 2000). Bending and torsional distortions in the human mandible follow the same behavior observed by Korioth and Hannam (1994). In sheep, the presence of both mandibular diastema and long mandibular body generates larger moment arms than in humans, in particular, accentuated transverse bending during ICP, sagittal bending during INC, and torsion during UNI clenching were observed. However, interestingly, the sheep mandibular body appears to be subjected to an inferior level of strain than the human mandibular body for all clenching tasks. This can be explained by morphological and size differences since our specific sheep mandible is more than twice in length and three times in volume compared to the human mandible. Our results show higher mechanical strains within the sheep mandibular diastema and, therefore, closer to the strains in humans, possibly due to the reduced mandibular diastema cross-sectional area and the higher lever effect of the sheep long mandibular body. Both the latter observation and the fact that sheep mandibular diastema has already been the subject of previous studies on fracture fixation (Gutwald et al., 2011; Schouman et al., 2016) led us to postulate that sheep mandibular diastema may present a more favorable mechanical environment compared to the sheep mandibular body.

We, therefore, reproduced two fracture scenarios in sheep, a mandibular body and a mandibular diastema fracture, to be compared to a human mandibular body fracture. The simple fractures were then fixated with two traditional, clinically used, parallel titanium 4-hole miniplates and monocortical screws in both human and sheep models. We found that tensile strains within the fracture gap in the human mandibular body were higher than in the sheep fractures. Notably, compressive strains in the sheep mandibular body fracture were higher than in humans during INC and UNI clenching, in the latter up to 86% higher. The mandibular diastema fracture showed always the lowest strains. Generally, the observed differences in the strain levels indicate that the same fixation system could lead to differences in the bone regeneration process. Previous studies have been able to predict bone healing in sheep (Claes and Heigele, 1999; Isaksson et al., 2006) and humans (Byrne et al., 2011) using the same levels of mechanical signal to drive the bone formation process. Our results show that, if bone regeneration in humans and sheep is regulated by the same level of mechanical signals (den Boer et al., 1999), a different healing outcome might be expected.

Peak von Mises stresses within the implants in the sheep mandibular diastema fracture were comparable or lower than in humans. On the contrary, during INC and UNI clenching, higher stresses were found within the implants in the sheep mandibular body fracture, thus increasing the risk of implant failure in this region. Furthermore, UNI clenching is not only the biting task leading to higher mechanical solicitations, but it is also known to be the most frequent biting task in sheep. Since one could expect that, post-operatively, sheep would not be able to consciously reduce the force and frequency of biting due to sedation and analgesia, therefore, a fortiori, the mandibular diastema region seems to be the safer choice for testing fixation devices.

There are several limitations in this study that need to be mentioned. The same clenching tasks were investigated for the human and the sheep mandible, however, the sheep grazing activity results not only in compressive but also in shear movements at the occlusion, not simulated here. It has been suggested that large compressive forces make grass leaves behave as brittle materials (Sanson, 2006) and therefore it can be expected that sheep apply lower forces during the subsequent translational biting with a consequent lower risk for fixation failure. Moreover, uncertainties in the estimation of the muscular force magnitudes in sheep must be considered. Since no experimental data is available about sheep’s maximum bite force, a sheep model verification was not possible. However, the forces predicted at the occlusion for sheep support the hypothesis that dolichocephalic mandibles produce weaker bite forces than brachycephalic ones (Ingervall and Thilander, 1974; Van Spronsen et al., 1992; Sella-Tunis et al., 2018), possibly due to relatively shorter moment arms of the muscles in longer mandibles (Throckmorton et al., 1980). Other limitations include the definition of linear elastic isotropic material properties of the bone tissues, with no distinction between teeth and cortical bone, and without considering the orthotropic properties of human cortical bone (Schwartz-Dabney and Dechow, 2003). We additionally tested the influence of orthotropic properties of the cortical bone in the model of the healthy human mandible and we modeled teeth according to the biomechanical properties reported by Lovald et al. (2010). We did not predict considerable changes in the mechanical strains (see Supplementary Data 1). In addition, teeth were modeled as a whole continuous structure, which may have led to increased stiffness of the mandibles, however, we did not see substantial differences in the predicted biomechanics compared to other studies (Korioth et al., 1992; Baek et al., 2012). Despite similarities in the trabecular architecture of human and sheep mandibles (Watson et al., 2018), no experimental values were found on Young’s modulus of sheep mandibular bone tissue, which was assumed similar to goats, whose values were calculated by Li et al. (2013) using the relationship between Hounsfield units, bone density, and elastic modulus. Previous studies in long bones have reported a great variability of elastic modulus values of cortical bone in the humans and sheep, 14–22 GPa (Keyak et al., 2005) and 15–32 GPa (Spatz and Vincent, 1996; Grant et al., 2014), respectively. In general, a relatively higher Young’s modulus in sheep cortical bone might be expected. We have performed additional simulations with higher Young’s modulus for sheep cortical bone and have observed slightly lower mechanical strains (see Supplementary Data 2) not influencing the conclusions drawn from this study. Future *in vivo* studies should focus on the validation of the sheep mandibular model by measuring the forces at the occlusion during different clenching tasks and correlating the resultant mechanical strains induced on the mandibular bone by both muscular forces and reactions at the occlusion.

In conclusion, we investigated the biomechanics of sheep mandible as a possible large animal model for preclinical studies on mandibular fracture fixation, as sheep have been often used to test fixation devices and to analyze the bone healing response (Claes and Heigele, 1999; Gutwald et al., 2011). We used finite element analysis to evaluate and compare the biomechanical behavior of the human and sheep mandibles in healthy and fractured conditions. In the healthy models, the mechanical strains within the sheep mandibular diastema were closer to humans, compared to the sheep mandibular body. Similarly, in the fractured models, despite lower strains at the fracture site, stresses within the implants in the sheep mandibular diastema were closer to the stresses in humans, i.e., the loading environments of the fixation systems were comparable. Higher implant solicitation was found in the sheep mandibular body region for specific clenching tasks.

These results suggest that, despite the clear anatomical differences, the sheep mandibular diastema may be a more suitable location for biomechanical evaluation of fracture fixation devices that are intended to stabilize human mandibular fractures. However, relevant differences in the strain magnitudes at the fracture site exist between human mandibular body fractures and sheep mandibular diastema fractures, which might lead to distinct healing responses *in vivo*.

## Data Availability Statement

The datasets presented in this article are not readily available because they present some patented elements. Requests to access the datasets should be directed to SC, sara.checa@charite.de.

## Author Contributions

CR, VO, GD, MH, and SC designed the study. HF performed the CT and MRI scans and the anatomical observations. VO developed the computational models and collected the data. VO and SC interpreted the data and drafted the manuscript. All authors read and revised the manuscript and approved its content.

## Conflict of Interest

The authors declare that the research was conducted in the absence of any commercial or financial relationships that could be construed as a potential conflict of interest.
