# Supplementary material for: Biomechanical Assessment of the Validity of Sheep as a Preclinical Model for Testing Mandibular Fracture Fixation Devices
Source: Front Bioeng Biotechnol. 2021 May 6;9:672176. doi: 10.3389/fbioe.2021.672176 (PMC8134672; doi:10.3389/fbioe.2021.672176)
Supplement: Supplementary file 1 [file Data_Sheet_1.DOCX]

Supplementary Material

# Supplementary Data 1

**Supplementary Figure 1.** Average maximum and minimum principal strains within the healthy human mandibular body during the intercuspal (ICP), incisal (INC), and unilateral (UNI) clenching tasks, in both working (WS) and balancing (BS) side, in case of isotropic and orthotropic (Lovald et al., 2010) cortical bone properties. In the latter case, dentin properties were assigned to teeth.

# Supplementary Data 2

According to the experimental values found in sheep long bones (Spatz et al., 1996), Young’s modulus of cortical bone was increased to 30,000 MPa in the sheep mandibular diastema fracture model. We found lower strains within the fracture gap (Supplementary Figure 2.1) as well as lower stresses within the implants (Supplementary Figure 2.2).

**Supplementary Figure 2.1.** Average maximum and minimum principal strains within the human mandibular body and sheep mandibular diastema fractures during the intercuspal (ICP), incisal (INC), and unilateral (UNI) clenching tasks, simulating an increased Young’s modulus (Y) of sheep cortical bone (light pink).

**Supplementary Figure 2.2.** Von Mises stresses within the top and bottom implants in the human mandibular body and sheep mandibular diastema fracture scenarios during the intercuspal (ICP), incisal (INC), and unilateral (UNI) clenching tasks, simulating an increased Young’s modulus (Y) of sheep cortical bone (light pink).
